# A *SRY*-HMG box frame shift mutation inherited from a mosaic father with a mild form of testicular dysgenesis syndrome in Turner syndrome patient

**DOI:** 10.1186/1471-2350-11-131

**Published:** 2010-09-19

**Authors:** Mohammad Shahid, Varinderpal S Dhillon, Hesham Saleh Khalil, Shameemul Haque, Swaraj Batra, Syed Akhtar Husain, LHJ Looijenga

**Affiliations:** 1 College of Dentistry, Alkharj University, Alkharj, Kingdom of Saudi Arabia; 2 Commonwealth Scientific and Industrial Research Organisation (CSIRO) Food and Nutritional Sciences, Gate 13, Kintore Avenue, PO BOX 10041, Adelaide SA 5000, Australia; 3 College of Dentistry, King Saud University, Riyadh, Kingdom of Saudi Arabia; 4 Department of Biosciences, Jamia Millia Islamia (A Central University), Jamia Nagar, New Delhi 110025, India; 5 Department of Obstetrics and Gynecology, Lok Nayak Jai Prakesh Hospital and Associates, Maulana Azad Medical College Campus, Bhadur Shah Zafar Marg, New Delhi 110002, India; 6 Department of Pathology, Erasmus MC - University Medical Center Rotterdam, Daniel den Hoed Cancer Center, Rotterdam, The Netherlands

## Abstract

**Background:**

Sex determining factor (SRY) located on the short arm of the Y chromosome, plays an important role in initiating male sex determination, resulting in development of testicular tissue. Presence of the *SRY *gene in females results in XY sex reversal and increased risk of gonadal germ cell tumours if the karyotype also includes the so-called GonadoBlastoma on the Y chromosome (GBY) region. The majority of mutations within the *SRY *gene are *de novo *affecting only a single individual in the family. The mutations within the high-mobility group (HMG) region have the potential to affect its DNA binding activity.

**Case Presentation:**

We performed G- and R-banding cytogenetic analysis of the patient and her family members including her father. We also performed molecular genetic analysis of *SRY *gene. Cytogenetic analysis in the patient (Turner Syndrome) revealed the mosaic karyotype as 45, X/46, XY (79%/21% respectively) while her father (milder features with testicular dysgenesis syndrome) has a normal male karyotype (46, XY). Using molecular approach, we screened the patient and her father for mutations in the *SRY *gene. Both patient and her father showed the same deletion of cytosine within HMG box resulting in frame shift mutation (L94fsX180), the father in a mosaic pattern. Histological examination of the gonads from the patient revealed the presence of gonadoblastoma formation, while the father presented with oligoasthenozoospermia and a testicular seminoma. The frameshift mutation at this codon is novel, and may result in a mutated SRY protein.

**Conclusion:**

Our results suggest that lack of a second sex chromosome in majority cells of the patient may have triggered the short stature and primary infertility, and the mutated SRY protein may be associated with the development of gonadoblastoma. It is of importance to note that mosaic patients without a SRY mutation also have a risk for malignant germ cell tumors.

## Background

Turner Syndrome (TS) is a relatively common chromosomal disorder, caused by complete or partial X monosomy in some or all cells [1]. Almost half of the cases have typical TS karyotype (45, X), whereas the remaining cases either have a derivative sex chromosome in the investigated cells or a mosaic karyotype, with the second cell line having a normal or structurally rearranged sex chromosome. TS with different karyotypes have demonstrated the presence of a Y-chromosome or Y- derived material in frequencies ranging from 4-61% [2,3]. TS with mosaic 45, X/46, XY karyotype comprises a phenotype spectrum of female (10-15%) having mutated SRY and an increased risk for developing of gonadoblastoma or dysgerminoma [4]. *SRY *is proven to direct sex-determination pathway towards male development [5,6]. This single exon gene is located on distal part of the short arm of Y-chromosome spanning 3.8 kb and encodes a 204 amino acid protein. *SRY *contains a DNA-binding domain high-mobility group (HMG) motif in the middle of the protein [5,7]. The presence of DNA-binding domain in SRY protein suggests its regulatory role and it could work as a transcription factor. Inactivating mutations in *SRY *gene cause failure to develop testis and has been found to account for approximately 15% of cases with gonadal dysgenesis and XY sex-reversal [8,9]. However, a majority of these patients may have mutations in other genes involved in sex determination and differentiation (in term for development of phenotypical sex characteristics) pathway or in the regulatory elements of the *SRY *gene. To the best of our knowledge more than 60 mutations have been identified within open reading frame (ORF) of the *SRY *gene, and majority of these are located within HMG box, thus highlighting the vital and significant role of this domain [10-12]. Less than 20 familial mutations in *SRY *gene have been reported so for in the literature. Here, we report a new point mutation in TS patient who inherited the mutation from a phenotypically normal father. However, this male showed a form of Testicular Dysgenesis Syndrome, i.e., oligoasthenozoospermia and a testicular seminoma. To the best of our knowledge no mutations at the above mentioned evolutionary conserved codon have been reported previously in the literature.

## Case Presentation

Informed consent was obtained from the patient, her family members and control individuals who participated in the present study. This study was approved by the ethics as well as bio-safety committee of the hospital and university.

### Patient II-5

Patient II-5, a 23 years old female, with short stature, ambiguous external genitalia and absence of pubertal development was referred for cytogenetic analysis. Physical examination revealed her height as 145 cm (below the fifth percentile). This patient showed multiple Turner stigmata such as micrognathia, low-set ears, high-arched palate, short and webbed neck, bilateral cubitus valgus, low hair line, presence of Müllerian structure, widely spaced nipples, nail dysplasia and multiple nevi. Sparse axillary and pubic hairs (Tanner stage II) were observed, and there was no clitoromegaly. Endocrinological studies demonstrated hypergonadotropic hypogonadism (estradiol 12 pg/ml; LH 26.2 mIU/ml; FSH 50.2 mIU/ml) as well as normal female concentrations of testosterone and androstenedione. Sonographic examination revealed a normal-sized uterus with a thin endometrium and bilateral Müllerian derivatives. Ovaries were not visible, but thickened structures resembling streak gonads were present. Histological investigations revealed only fibrous stromal tissue. Gonadoblastoma was found in the streak gonads. Standard cytogenetic analysis was performed and showed mosaic 45, X/46, XY karyotype.

### Patient I-1

**Figure 1:**
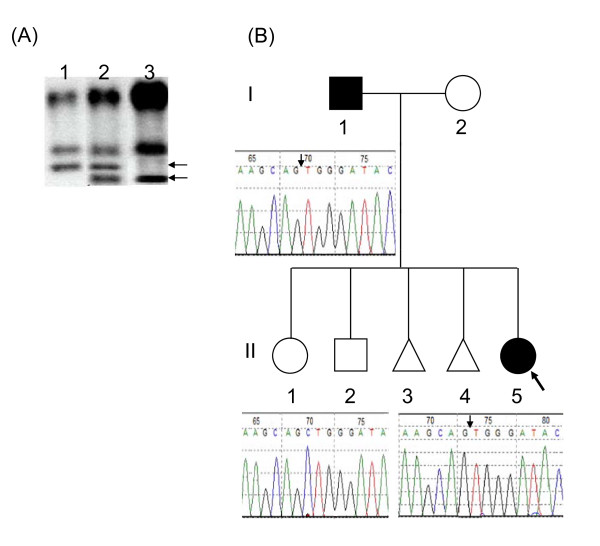
**Mutation(s) in the SRY gene in the affected family**. (A) Polymerase chain reaction-SSCP analysis of *SRY *gene. Lane 1, *SRY *from normal male control DNA (II-2); lanes 2 and 3: *SRY *with altered band from patient I-1 and II-5 respectively. The migration pattern from I-1 clearly indicates that there are two different alleles that he shares with normal son (not shown) and affected daughter; (B) Pedigree of the affected family also showing partial electropherograms of the mutation (deletion of C in the HMG box (helix 2) leading to a premature stop codon as L94fsX180) identified in patient II-5 and her father (I-1). These results were obtained by sequencing of the amplification products after cloning. Wild-type electropherogram identified in the normal brother (II-2) is also indicated. White symbols denote unaffected individuals where as black symbols denote affected individuals. The arrow indicates the proband (II-5).

He is the father of patient II-5 (Fig. 1). At the age of 53 yr, his height and weight were 1.79 m and 84 kg respectively. The patient had normal secondary sex characteristics. Orchidectomy was performed at the age of 48 yr for the presence of pure testicular seminomatous tumor. Histological examination of the gonad did not present any signs of dysgenesis. At the time of examination, his plasma testosterone (4.1 ng/ml; normal 2.5-9.5) and LH (3.9 mU/ml; normal 2.2-5.8) and FSH levels (7.1 mU/ml; normal 2-10) were within the normal range of an adult male. Semen analysis showed signs of oligoasthenozoospermia (volume 1.4 ml; semen concentration 7.4 × 10^6^/ml; normal 20-40 × 10^6^/ml; 37% motile sperm after 1 h). Reduced semen quantity and presence of testicular cancer classifies him as the one with milder form of testicular dysgenesis syndrome.

### Cytogenetic and Molecular analysis

Both G- and R-banding was performed on metaphase spreads from peripheral blood of the patient, her siblings and parents. Genomic DNA was extracted from peripheral blood as well as gonadal tissue using DNA isolation kit following manufacturers' instructions. SRY polymerase chain reaction amplifications, single strand conformational polymorphism and sequencing were performed as reported elsewhere [13]. The resulting PCR products from I-1, II-5 were cloned and then 10 clones were sequenced. In present study, cytogenetic analysis has been done using G- and R-banding technique. The patient (II-5) had a karyotype 45, X/46, XY (the ratio being 79% and 21% respectively) and her father (I-1) a normal male karyotype (46, XY, 100%). Patient II-5 showed altered level of estradiol, LH, FSH but with normal female concentration of testosterone and androstenedione. Both patient and her father showed altered migration of PCR products in the SSCP assay (Fig 1A). Both direct sequencing and sequencing from cloning revealed a point mutation in HMG box of SRY gene in the patient (Fig 1B). The sequence has a deletion of C (cytosine) leading to frame shift mutation within the open reading frame inside the highly conserved DNA-binding motif-HMG box leading to a premature stop codon. The mutation is described as L94fsX180. Sequencing of both PCR products and cloning products confirmed the presence of the same mutation as seen in his daughter (II-5) however, the mutation is mosaic in nature. It is clear from the pedigree as well that gonadal mosaicism exits as indicated by the affected daughter and the normal son, which were genetically proven to be the biological children of the father.

**Figure 2:**
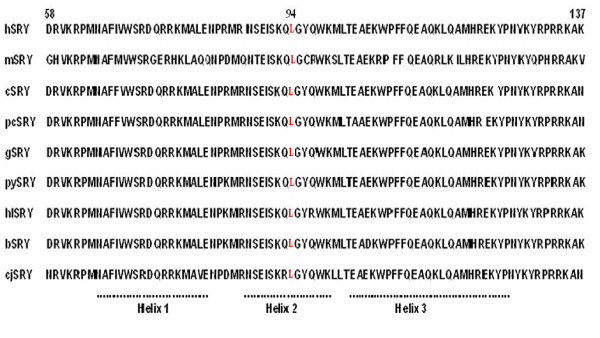
**Alignment of HMG box sequences of SRY proteins from different mammalian species**. Position 94 (shown in red) is highly conserved in different species (h: human; m: mouse; c: Chimpanzee; pc: Pygmy Chimpanzee; g: gorilla; py: Pongo; hl: Hylobates; b: Baboon and cj: Calitrix).

Normal male sex determination in mammals is targeted by the *SRY *gene present on Y chromosome. Its timing and expression is exquisitely regulated and must probably reach the required threshold for testis formation in the developing embryo [10]. The patient reported here have a mosaic 45, X/46, XY karyotype with multiple Turner stigmata. We identified a new *SRY *mutation in this patient. This mutation, leading to a premature stop, may result in altered non-functional protein as evident by the inability to develop male sex organs. Sex determination during early embryonic stages is characterized by complex interaction of various genetic and non-genetic factors [14]. There is significant evidence that *SRY *is essential for sex determination [5]. However, there are examples of *SRY*-negative individuals who differentiate to males (20% XX males). Mutations in *SRY *gene and presence of the dominant 45, X cell line in the patient may have acted in a cumulative manner to induce a cause-effect relationship. This may account for non-masculinization in an otherwise originally XY embryo. Unequal distribution of two cell lines as seen in this patient may have originated at the time of implantation and their distribution into fetal and placental poles [15]. SRY protein belongs to the SOX family of transcription factors characterized by HMG domain having DNA binding and bending properties. It has the ability to mediate protein-protein interactions and contain signals for its nuclear import [16,17]. This mutational change as reported here in this report may have an electrostatic and hydrophobic interaction with phosphate and sugars, respectively, of the DNA backbone. This alteration can result in specific orientation and binding DNA bases in the major groove which can thus totally or at least partially inhibit or reduce its (SRY) interactions with DNA [18]. Deletion of cytosine at codon 94 which is evolutionary conserved (Fig. 2) in mammals has resulted in a nonsense frame shift mutation (L94fsX180) within the HMG box, resulting in an altered protein at the C terminus end. A phenotypical female with mosaic 45, X/47, XYY karyotype and a frame-shift mutation at codon 4 of *SRY *gene has been reported in the literature [19]. It has been shown that the mutant *SRY *may be assumed to induce a non-functional SRY-coded protein that lacks DNA-binding motif. Similarly there is another report that describes two patients with 45, X/46, XY (mosaic karyotype) have a missense mutation as S18N in the 5' non-HMG box region in blood as well as in streak gonads [20]. Mutations in *SRY *gene are among known causes of 46, XY pure gonadal dysgenesis (PGD). It has been generally accepted that mutations in HMG box disrupt the gene's function. Likewise, mutations outside HMG box have been reported in several patients with PGD, and one has been detected in a 46, XY female with partial ovarian function [20-22]. We analyzed DNA from her father and found the similar mutation but in the mosaic condition in peripheral blood as well as in sperm DNA. Transmission of this mutation from father to the affected daughter and a wild type *SRY *sequence to the normal son further provide the basis that this mutation is present in the mosaic condition in father. The mutation has resulted in a truncated SRY protein and hence, could have altered its binding activity. The development of normal male sex determination and differentiation might be due to the presence of a transcript with wild type SRY protein due to the mosaicism in the gonadal tissue. It is quite possible that some mutational event(s) might have occurred during the early embryonic life. Our findings are further strengthened by other reports of transmission of mutation from fertile fathers to their XY daughters [14,23,24]. In the present study, the patient also developed a bilateral gonadoblastoma, a precursor lesion for malignant germ cell tumors in the dysgenetic gonad is further supported by previous studies [25-29].

It is widely recognized that dysgenetic gonads, in some cases related to *SRY *mutations develop gonadoblastoma only in case GBY region is present in the genome. However, the molecular cause of gonadoblastoma formation remains elusive. The failure of indifferent gonads to develop during embryogenesis into testes ultimately leads to a phenotypical female with delayed puberty and amenorrhea. The GBY locus on Yq region is thought to contain a proto-oncogene involved in the origin of these tumors [30], for which *TSPY *is one of the likely candidates, supported by strong expression of TSPY in CIS and GB [31]. Normally, TSPY is expressed in spermatogonia of the adult testis and is believed to be related to mitotic proliferation [32]. The presence of streak gonads may be attributed to the invasion of primary genital ridge by the 45, X cell line during early developmental stages. Pubertal virilization in some TS patients represents an alarming sign of undetected Y-chromosome positive cell lines that increase risk for developing gonadoblastoma [19]. The mosaicism with Y-bearing cell line (though with defective/mutated *SRY*) even at low levels formed during paternal meiosis and maternal non-disjunction during meiotic process may explain the mechanisms underlying the abnormal sex development. This is the second report that describes the mosaic fertile father who shows milder features associated with testicular dysgenesis syndrome and third report that describes the patients with sex development disorders (DSD) having mosaic mutation in *SRY *gene [24,33]. The risk for the development of germ cell tumors is an important factor to deal with the management of patients having sex development disorders (DSD) [34]. To our knowledge this is the first report that describes the *SRY *mutation in father who also shows oligoasthenozoospermia and has developed testicular seminoma. Both oligoasthenozoospermia and testicular cancer comprises the milder form of underlying entity associated with Testicular Dysgenesis syndrome (TDS) which can be caused by genetic and/or environmental factors. However, till date no specific causes of this entity have been identified. It may be possible that insufficient androgen production during the fetal testis developmental stages may be associated with downstream entities like TDS. It may be noted that the grand father (deceased) of this patient (II-1) was associated with the pesticide industry for quite a long time before the birth of patient I-1. Therefore, it is possible that this environmental exposure may also be responsible to certain extent for this condition in I-1 that is inherited by the patient II-5. The present and the previous published paper [24] strengthens the role various genetic factors along with the underlying mutations in *SRY *gene play in abnormal sex development disorders, related to malignant transformation of germ cells.

## Conclusion

The present finding, especially the frameshift mutation in the highly conserved codon in the HMG box of *SRY *gene, further strengthen the functional importance of this gene in the sex development. To the best of our knowledge this is the first case (I-1) with the variant form of TDS phenotype having mosaic mutation in *SRY *gene. The frameshift mutation has been inherited by the daughter (II-5). It is therefore, concluded that TS patients must be analysed both by conventional cytogenetic and molecular genetics approaches to rule out the presence of the Y chromosome and/or the *SRY *gene, as well as the GBY region.

## Competing interests

The authors declare that they have no competing interests.

## Authors' contributions

VSD and SAH participated in the conception and the design of the study, MS conducted the molecular analyses, SH performed cytogenetic analysis, HSK performed endocrinological analysis and SB clinically diagnosed the family. VSD and LHJL have written the manuscript while others helped in further revising the manuscript. All authors read and approved the final manuscript.

## Pre-publication history

The pre-publication history for this paper can be accessed here:

http://www.biomedcentral.com/1471-2350/11/131/prepub
